# Motivational Underpinnings of Fluid Reasoning: The Fit Between Motivational Orientation and Task Demands in Promoting Psychological Flow

**DOI:** 10.3390/jintelligence14080167

**Published:** 2026-08-01

**Authors:** Alessio Tesi

**Affiliations:** Department of Political Science, University of Pisa, 56126 Pisa, Italy; alessio.tesi@unipi.it

**Keywords:** flow, fluid intelligence, motivation, prevention, promotion, regulatory fit, regulatory focus

## Abstract

The present study examined how different combinations of promotion and prevention focus are associated with the experience of flow during fluid reasoning tasks of different difficulty, and whether flow is in turn associated with fluid intelligence assessed through a fluid reasoning task. Promotion focus reflects a self-regulatory orientation toward ideals, advancement, and gains, whereas prevention focus reflects an orientation toward duties, security, and non-losses. A total of 242 participants completed self-report measures of regulatory focus and task-related flow, along with a fluid reasoning task. One group completed a lower-demanding version of the task (*N* = 136), whereas the other completed a higher-demanding version (*N* = 106). A three-way moderated mediation analysis was conducted to test the indirect association between a promotion focus and fluid reasoning, mediated by flow, at the conditional level of high versus low levels of prevention for each difficulty condition. The results showed that, in the lower-demanding condition, promotion focus was more strongly positively associated with flow when prevention focus was low rather than high. In the higher-demanding condition, promotion focus was more strongly positively associated with flow when prevention focus was high rather than low. Across both conditions, higher flow was associated with better performance in the fluid reasoning task. Overall, the findings suggest that different motivational configurations may be differentially compatible with fluid reasoning task demands, and that flow may represent one process through which motivational orientations are associated with fluid intelligence.

## 1. Introduction

### 1.1. Motivation and Fluid Reasoning

Across the major facets of human intelligence, fluid intelligence has been considered one of the central human intellectual abilities referring to the capacity to solve new problems, detect relationships, abstract rules, and infer underlying structures relatively independently of previously acquired knowledge (Cattell, 1987). In this sense, fluid intelligence can be distinguished from forms of intelligence that rely more heavily on crystallized knowledge stored in long-term memory (Schneider & McGrew, 2018). Fluid intelligence is also considered a domain-general ability, as it supports reasoning across different types of content rather than within a single knowledge domain. A large body of research suggests that fluid intelligence is closely associated with working memory and executive functioning, including the maintenance and manipulation of information, attentional control, and inhibition of irrelevant responses (i.e., Blair, 2006; Hagemann et al., 2023; Stocco et al., 2021). This ability is especially important for adaptation and learning, and it predicts performance in cognitively demanding contexts such as school, higher education, and work (Schneider & McGrew, 2018). Among the available measures used to assess fluid intelligence, Raven’s Progressive Matrices (J. Raven, 2008) are one of the most widely used instruments. More specifically, they are non-verbal matrix reasoning tasks designed to assess fluid reasoning (FR), a central and widely recognized indicator of fluid intelligence. Progressive Matrices require respondents to identify patterns and infer the underlying rules that govern the structural relations among visuospatial elements. Accordingly, they assess relational reasoning, namely the ability to detect and integrate one or more relations between mental representations (E. Ferrer et al., 2009). Moreover, Raven matrices are characterized by increasing item difficulty: as the complexity of the patterns and response alternatives increases, items become more difficult to solve. This structure makes the test particularly suitable for capturing individual differences in fluid reasoning ability, which in turn provides an important indicator of fluid intelligence.

Despite the centrality of fluid intelligence in human cognition, most research has predominantly examined the cognitive architecture of fluid intelligence. By contrast, less attention has been devoted to motivational variables that may shape the conditions under which these cognitive resources are optimally deployed.

Human motivation is a driving force that initiates, directs, and sustains goal-directed behavior, and it represents a crucial determinant of individuals’ performance and engagement in specific tasks. In cognitively demanding activities such as those that require fluid intelligence (Hagemann et al., 2023), motivation may influence the extent to which individuals effectively deploy cognitive resources such as attention regulation, persistence, and working memory, all of which are relevant for fluid intelligence (Kanfer & Ackerman, 1989). More broadly, research has shown that the interplay between cognitive ability and motivation is closely related to task performance and engagement in goal-directed activities (Van Iddekinge et al., 2018).

Motivation can be conceptualized at both dispositional (i.e., trait-like) and situational levels (i.e., state-like) (Van Iddekinge et al., 2018). At the dispositional level, individuals may differ in their relatively stable tendencies to pursue goals with effort and persistence. In this respect, Regulatory Focus Theory (Higgins, 1997; Scholer & Higgins, 2012) distinguishes between two motivational systems that reflect different self-regulatory concerns: promotion focus, which is oriented toward growth, advancement, and accomplishment, and prevention focus, which is oriented toward safety, security, and the fulfillment of duties and obligations. These orientations can be understood as reflecting differential sensitivity to distinct needs, such as nurturance versus security, and they shape how individuals approach goal pursuit (Higgins, 1997; Morris et al., 2022). At the situational level, motivation may also take the form of task-specific experiential states that sustain involvement in ongoing activity. One particularly relevant state is psychological flow, namely a state of deep absorption, intense concentration, and intrinsic enjoyment during task engagement (Csikszentmihalyi, 1990; Martin & Jackson, 2008).

Whereas prior research has examined the effects of regulatory focus on learning strategies, creativity, speed–accuracy trade-offs, and performance (Nakkawita & Higgins, 2024), and a separate body of research has linked flow to performance and cognitive engagement (Harris et al., 2023), little is known about how dispositional motivational orientations shape the experience of flow during fluid reasoning tasks, with potential consequences for fluid intelligence performance (Payne et al., 2011). This issue is particularly relevant given that fluid intelligence is only partially explained by basic cognitive functions, such as working memory, suggesting that motivational and experiential factors may also contribute to performance in reasoning tasks (Hagemann et al., 2023). Building on this rationale, the present study aims to test whether promotion and prevention orientations interact differently as a function of FR task difficulty, and whether these motivational patterns are associated with performance through increased task absorption and optimal engagement (i.e., flow). The following sections unpack the study hypothesis by examining the literature on the interplay between motivation and fluid intelligence. This involves addressing the role of promotion focus, prevention focus and psychological flow, while also considering task difficulty as a key factor that may influence the relationship between motivational patterns and fluid intelligence performance.

### 1.2. Regulatory Focus, Flow and Fluid Intelligence

Regulatory Focus Theory (Higgins, 1997) posits that goal-directed behavior is regulated by two coexisting motivational orientations, namely promotion focus and prevention focus. These orientations begin to develop early in life as self-guides that orient individuals toward the fulfillment of distinct needs. More specifically, promotion focus is rooted in nurturance and growth needs, whereas prevention focus is rooted in security and safety needs. Accordingly, promotion-focused individuals are especially sensitive to gains versus nongains and are motivated by advancement, accomplishment, and positive change, whereas prevention-focused individuals are especially sensitive to non-losses versus losses and are motivated by security, responsibility, and the avoidance of errors or failures (R. A. Ferrer et al., 2017; Scholer & Higgins, 2012). Consistent with this distinction, promotion focus is typically associated with eager strategic inclinations, openness to opportunities, and a broader, more exploratory mode of processing, whereas prevention focus is associated with vigilance, careful scrutiny of details, and a stronger concern with accuracy and error prevention (Scholer & Higgins, 2012).

Regulatory Focus Theory conceptualizes promotion and prevention as distinct, orthogonal systems rather than as opposite ends of a single continuum, such that individuals may be high or low on both dimensions (Scholer & Higgins, 2012). Although one orientation may be more salient at a given moment, both systems may jointly contribute to self-regulation in specific goal pursuits. Studies have suggested that the two foci can coexist within the same person rather than being mutually exclusive (i.e., R. A. Ferrer et al., 2017; Haws et al., 2010; Nakkawita & Higgins, 2024; Petrou et al., 2017). Research supports the notion that different regulatory focus profiles can be observed. For example, individuals could be high or low in both focuses, or high in one and low in the other (Förster et al., 2003). This supports modeling the two systems simultaneously and examining their potential interaction when theoretical predictions concern their combined contribution (i.e., Chen et al., 2017; Parker et al., 2014).

Previous studies have shown that regulatory focus influences how individuals approach task performance (Scholer & Higgins, 2012). For example, Crowe and Higgins (1997) found that promotion focus was associated with eagerness and a greater willingness to prioritize advancement, even at the risk of committing more errors, whereas prevention focus was associated with strategic vigilance and greater concern with accuracy, even at the cost of slower performance. Thus, promotion and prevention may foster different modes of engagement with the same task. Beyond these individual tendencies, task characteristics may also make one orientation, or a specific combination of both, more functional than another. For instance, when people face a highly demanding task, an efficient approach could be to employ systems that prioritize goals of advancement and accomplishment while preventing errors (Scholer & Higgins, 2012). This possibility is consistent with Regulatory Fit Theory, according to which individuals experience stronger engagement when the manner of the goal pursuit—the use of eager or vigilance strategies—fits (vs. unfits) their underlying motivational orientation, that is promotion and/or prevention (i.e., Barthelmäs & Keller, 2021; Higgins, 2005; Tesi et al., 2024).

The present study consistently examines whether motivational orientations are associated with better performance on an FR task. Specifically, the study hypothesized that a combination of high promotion and high or low prevention focus would promote greater engagement in FR tasks depending on task difficulty.

When a FR task is adequately challenging but not too high in difficulty (i.e., a lower-demanding FR task), a combination of a high promotion focus and a low prevention focus is associated with better task engagement. A promotion focus ensures an eager, advancement-oriented approach that aligns with how a lower-demanding FR task is likely processed (Lee & Higgins, 2009). However, the vigilant mode of prevention, which aims to ensure security, accuracy, and the avoidance of mistakes, is less functional due to the task characteristics. In fact, if activated, it would introduce a slower responding or reduce fluidity of engagement due to the high propensity for scrutiny and continuous assessment. By contrast, when FR tasks are harder (i.e., higher-demanding FR task), involving more complex relational patterns to solve, so both motives of promotion focus (i.e., gain, advancement) and prevention focus (i.e., security, non-losses, prevent errors) may become functional. Under such conditions, a high presence of both focuses best fit the way a complex task should be processed, i.e., in a concrete and scrupulous manner (Lee & Higgins, 2009). This may be especially beneficial for task engagement. Thus, the functionality of different interactive configurations of regulatory systems can depend on task difficulty; for lower-demanding tasks the activation of the only promotion focus is optimal, while for high-demanding tasks both motivational approaches become functional. In the present study, task engagement is operationalized through the construct of psychological flow. Therefore, flow is considered a potential mediator of the relationship between motivational orientation and fluid intelligence performance.

### 1.3. The Mediational Role of Psychological Flow Between Motivational Orientations and Fluid Intelligence Across FR Task of Different Difficulty

Flow is a psychological state characterized by deep absorption, intense concentration, and intrinsic enjoyment during task engagement (Csikszentmihalyi, 1990). Although individuals may differ in their dispositional tendency to experience this optimal state, flow is primarily conceptualized as a state experience that occurs during the course of action (Norsworthy et al., 2021). Consistent with this perspective, flow can be optimally measured in situ contingent on a specific experience (Barthelmäs & Keller, 2021). In its classic formulation, flow encompasses a combination of interrelated characteristics, including a perceived balance between skills and challenges, clear goals, unambiguous feedback, concentration on the task at hand, a sense of control, reduced self-consciousness and concern about performance, altered time perception, and the experience of the activity as intrinsically rewarding (Jackson & Csikszentmihalyi, 1999).

Concerning possible antecedents, flow is closely related to motivational mechanisms and may be conceived as a regulatory compatibility experience (Keller & Bless, 2008), that is, as a state that is more likely to emerge when the way individuals are oriented to act matches the demands imposed by the task (Barthelmäs & Keller, 2021). For example, several studies have shown that when individuals’ skills match task challenges, the activity tends to be experienced as more intrinsically rewarding and engagement increases (Csikszentmihalyi, 1990; Landhäußer & Keller, 2012). However, this form of compatibility may not be limited to the fit between skills and task demands but may also involve a broader compatibility between personal (i.e., orientations, dispositions) and situational factors (Barthelmäs & Keller, 2021; Keller & Bless, 2008).

In the present study, flow is framed as an experiential state arising from person–task fit, that is, as a state that is more likely to occur when individuals’ dispositional motivational orientations are aligned with, and therefore functional for, the specific demands imposed by the FR task.

When individuals experience such a fit, they may be more willing to remain engaged in the activity, thereby increasing the likelihood that the task will be experienced as enjoyable and intrinsically rewarding. From this perspective, promotion and prevention focus are not expected to contribute uniformly to flow, but rather as a function of task difficulty. Promotion is associated with an eager, advancement-oriented approach to engagement, whereas prevention is associated with a vigilant, error-avoidant approach to engagement. Thus, when task demands are relatively lower, flow may be facilitated by a promotion-based mode of engagement. The way the lower-demanding FR task is processed (i.e., with more emphasis on advancement and less scrutiny) may be more compatible with a promotion focus. However, when task demands are higher, flow is more likely to occur when both motivational systems contribute interactively, because the manner in which the task is processed is compatible with both desired end states of promotion (i.e., gain) and prevention (i.e., non-losses) focus.

In terms of the potential consequences of the flow state on cognitive abilities, although there is relatively little direct evidence of the relationship between flow and cognitive performance (Barthelmäs & Keller, 2021), scholars have suggested that flow may involve cognitive processes such as focused attention, sustained concentration and low cognitive resource depletion (Landhäußer & Keller, 2012). Given that fluid reasoning performance relies, among other processes, on the ability to reallocate attention in response to self-generated feedback during problem solving (Stocco et al., 2021), the deep task-focused concentration experienced during flow may contribute to better performance on fluid reasoning tasks. For example, in FR tasks such as Raven-type matrices, successful performance requires individuals to monitor whether their current strategy is productive and, when necessary, disengage from previously attended but uninformative features in order to redirect attention toward more relevant aspects of the problem (Stocco et al., 2021). Because flow is characterized by intense task absorption, reduced self-consciousness, and a sense of effortless control (Jackson & Csikszentmihalyi, 1999), it may support reasoning by reducing self-focused evaluative monitoring and other forms of self-referential interference, while preserving, or even facilitating, the sustained task-oriented monitoring required for complex problem solving. Importantly, this reduction concerns self-focused evaluative processes rather than task-related cognitive control. Thus, prevention may remain compatible with flow insofar as it promotes vigilant scrutiny of task-relevant information rather than ruminative self-monitoring. However, only limited evidence has directly addressed the relationship between flow and fluid intelligence-related abilities (Payne et al., 2011; Ullén et al., 2012), and further research is needed.

Based on the assumption that flow represents an immersive and intrinsically rewarding state that supports task engagement, the present study hypothesizes a positive association between flow and fluid intelligence performance measured through an FR task.

### 1.4. The Present Study

The present study assumes that FR task difficulty may help clarify which motivational configuration is more functionally aligned for dealing with FR task of different difficulty. In particular, when the FR task is adequately challenging but not very difficult (i.e., lower-demanding FR task), successful performance may depend less on security-related concerns (i.e., constant vigilant monitoring aimed at avoiding losses/errors) and more on an advancement-oriented approach (i.e., eagerness to attain positive outcomes solving the task). Thus, a promotion-based orientation may be more compatible with the lower-demanding task (Barthelmäs & Keller, 2021; Keller & Bless, 2008). This allows individuals to pursue promotion-related goals, such as advancement, accomplishment, and gain, through eager means. In this condition, individuals may experience regulatory fit (Higgins, 2005; Lee & Higgins, 2009) and thus feel right about what they are doing, which may facilitate flow (Keller & Bless, 2008). Conversely, high prevention may be less compatible with a lower-demanding FR task, because heightened vigilance in this condition may introduce unnecessary monitoring and interfere with the smooth, intrinsically rewarding involvement that characterizes flow.

Because flow reflects a state of optimal involvement that is expected to support sustained engagement during task execution, it was further hypothesized that flow would be positively associated with FR scores.

**Figure 1:**
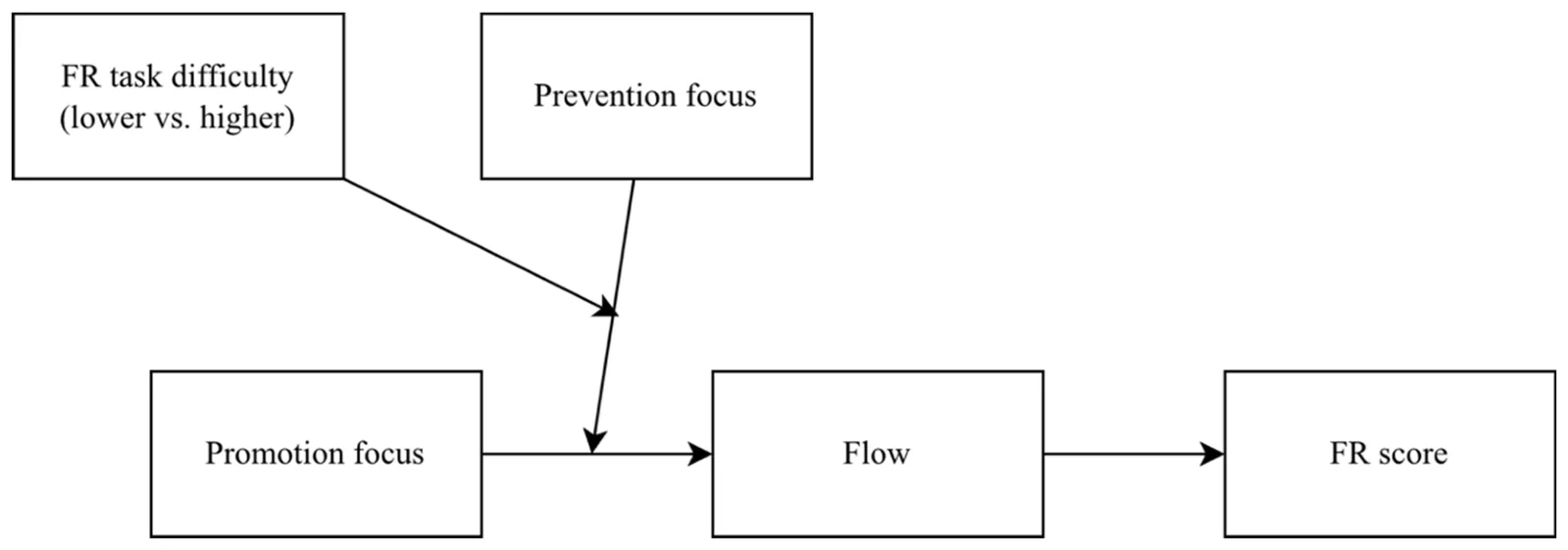
Study’s model.

On the other hand, when people are confronted with a difficult FR task (i.e., higher-demanding FR task), both advancement and security may become important. Performance may require not only eagerness to advance in the task, but also vigilance to monitor details and avoid negative outcomes. Under these more demanding conditions, promotion and prevention may jointly contribute to task engagement: promotion may sustain advancement and persistence in the face of high demands, whereas prevention may support careful checking, accuracy, and protection against mistakes (Scholer & Higgins, 2012). Thus, for difficult FR tasks, the most functional motivational configuration may not be a predominantly promotion-based mode alone, but rather the interactive contribution of both systems. Accordingly, the present study supports the hypothesis that, in the case of highly demanding FR tasks, flow is more likely to occur when both motivational orientations are jointly activated, such that the desired end-states associated with promotion and prevention are more compatible with the more scrupulous manner in which a higher-demanding FR task is processed. As in the previous case, flow was expected to be positively associated with FR scores. Accordingly, a three-way moderated mediation analysis was modeled (Figure 1).

In the present study, promotion and prevention focus were modeled as independent variables, whereas flow was conceived as a state-like motivational experience that may emerge during task engagement. This ordering was theoretically guided by regulatory focus theory, which conceptualizes promotion and prevention as self-regulatory orientations that may operate as relatively stable individual differences (Nakkawita & Higgins, 2024). By contrast, flow is commonly defined as a situational experiential state that emerges during ongoing task engagement, under conditions such as challenge–skill balance, clear goals, and unambiguous feedback (Jackson & Csikszentmihalyi, 1999; Norsworthy et al., 2021). Accordingly, FR performance was modeled as the final outcome of a motivational process linking trait-like regulatory orientations to state-like task engagement under different levels of task difficulty. The study hypotheses were as follows.

**Hypothesis** **1.** *In the condition of a lower-demanding FR task, promotion focus will be strongly positively associated with flow when prevention focus is low compared to high, and in turn, flow will be positively associated with FR scores*.

**Hypothesis** **2.** *In a higher-demanding FR task, a promotion focus will be strongly and positively associated with flow when a prevention focus is high compared to low. In turn, flow will be positively associated with FR scores*.

## 2. Method

### 2.1. Procedure and Participants

An a priori power analysis was conducted to guide data collection and estimate the sample size required to test the study’s focal hypothesis. Because the focal prediction concerned the unique contribution of the three-way interaction in explaining variance in flow, sample size planning targeted the local effect (incremental R^2^) associated with this interaction term. G*Power 3.1 was used, adopting a procedure specifically designed for regression-based power analyses of incremental explained variance (Faul et al., 2009). Accordingly, the analysis specified one tested (focal) predictor, the three-way interaction term, within a regression model including seven predictors in total. An alpha level of 0.05 and a target power of 0.90 were adopted, reflecting a relatively conservative criterion against type II error. The target local effect size was set at *f*^2^ = 0.05. This value was selected as a plausible small-to-moderate incremental effect, being larger than Cohen’s conventional benchmark for a small effect (*f*^2^ = 0.02) while remaining below the benchmark for a medium effect (*f*^2^ = 0.15) (Cohen, 1988). This choice is also consistent with methodological evidence indicating that interaction effects are often modest in magnitude and tend to explain limited incremental variance beyond lower-order effects (Sommet et al., 2023; Van Iddekinge et al., 2018). Under these assumptions, the required total sample size was *N* = 213.

According to the power analysis, a convenience sample of 242 participants were recruited. Participants were recruited through a non-probability snowball sampling procedure. The initial recruitment pool consisted of Psychology students, who were invited to complete an anonymous self-report questionnaire and to further distribute the study link through their personal and online social networks. Additional recruitment was conducted through online participant-recruitment platforms. The empirical sample was therefore based on voluntary participation. Participants gave explicit informed consent. All participants were Italian nationals, 102 were male, and 140 were female. The mean age was 25.81, and the educational status was the following: 151 had a high-school degree, 87 were college graduates and 4 had a PhD/Master of Professional Studies. Participants were randomly assigned to two conditions: a first group (*N* = 136) completed a lower-demanding FR reasoning task of balanced difficulty, and a second group (*N* = 106) completed a significantly more difficult FR task (see the “Measures” section). Immediately after completing the FR task, participants completed the Short Flow Scale, and finally participants’ motivational orientations were measured.

### 2.2. Measures

*Regulatory focus.* For measuring the promotion and prevention systems an adapted version of the Italian version of the Regulatory Focus Questionnaire (Higgins et al., 2001) was used. The scale was composed of 9 items (i.e., five items for promotion: “Do you often do well at different things you try?”; four items for prevention: “Not being careful enough has gotten me into trouble at times”). The response format was on 5-point Likert scale ranging from 1 = “never or seldom” to 5 = “very often”. A composite score was calculated by averaging across responses to the items. Promotion scale Cronbach’s α = 0.61; prevention scale Cronbach’s = 0.70.

*Flow*. The experience of flow during the FR task was assessed using the Italian version of The Short Flow Scale (Martin & Jackson, 2008). In order to best capture the flow state, the scale was administered immediately after the completion of the FR task. The instructions reminded participants to fill out the scale thinking about “your feeling during the completion of the previous task”. The scale was composed of 9 items on 7-point Liker scale (1 = totally disagree; 7 = totally agree) intercepting the foundational characteristics of flow, namely, challenge skill-balance, action-awareness, clear goals, unambiguous feedback, concentration on the task, sense of control, reduced self-consciousness, time transformation and autotelic experience (i.e., “I am completely focused on the task at hand”). A composite score was calculated by averaging across responses to the items. Cronbach’s α = 0.84.

*Fluid intelligence.* Fluid intelligence was assessed using a FR task based on the structure of Raven’s Progressive Matrices (J. C. Raven, 1956). The test presented participants with several matrices, each of which had a pattern from which a piece was missing. Participants were asked to select a piece from several alternatives that best resembled the configural pattern, which was governed by underlying abstract and logical rules. This assessed reasoning by analogy. As documented in the literature, visuo-spatial matrices are highly diagnostic of fluid intelligence (J. Raven, 2008; Zurrin et al., 2024). In the original Raven’s Progressive Matrices, items are organized into sets (A, B, C, D, and E) that progressively increase in difficulty. This progression reflects increasing relational complexity: participants must identify relevant visual-spatial features, infer one or more relations among them, and apply these relations to select the missing element. Accordingly, more difficult items place greater demands on relational reasoning, a core component of fluid intelligence.

Two FR task versions differing in overall demand level were constructed. The lower-demanding version was intentionally composed of items spanning a broader range of complexity, rather than exclusively easy items, in order to avoid a trivial task version that might have reduced meaningful variability in task engagement and, consequently, in the experience of flow. The higher-demanding version was composed predominantly of items classified as medium or high in complexity. Depending on item complexity, each matrix was followed by six or eight response alternatives. Participants received 1 point for each correct answer and 0 points for each incorrect answer. No time limit was imposed.

To obtain preliminary psychometric support for the FR tasks used in the present study, a dichotomous Rasch model using Jamovi software version 2.6 (The Jamovi Project, 2025) was fitted to the item responses, an approach appropriate for binary-scored items that allows item difficulty and item fit to be examined separately (Rasch, 1960; Wright & Linacre, 1994). For the lower-demanding form, the initial 15-item solution showed one clearly anomalous item (item 13) which combined an extremely low proportion of correct responses (0.007) with severe misfit (outfit = 19.264), indicating that it did not function consistently with the remaining items. A refined 14-item lower-demanding form excluding item 13 was therefore retained. In this revised version, item difficulty was more coherent, with proportions correct ranging from 0.118 to 0.985, and item-fit statistics fell within a substantially more homogeneous range (infit = 0.811–1.144; outfit = 0.199–1.546), with person reliability remaining limited but comparable to the original version (from 0.535 to 0.508). For the difficult form, the initial 15-item solution showed item-level problems for items 7, 4, and 8, which combined very low proportions of correct responses (0.057, 0.038, and 0.123, respectively) with elevated outfit values (2.321, 1.540, and 1.445), suggesting that these items were not simply difficult but also unstable. A refined 12-item difficult form excluding items 4, 7, and 8 was therefore retained. In the revised difficult version, item difficulty remained appropriately high, with proportions correct ranging from 0.104 to 0.953, while item-fit statistics were concentrated in a narrower and more acceptable range (infit = 0.910–1.166; outfit = 0.872–1.292), with a modest improvement in person reliability relative to the original solution (from 0.534 to 0.600). The full Rasch analyses are presented in Supplementary Material. Taken together, these analyses were intended to refine both task versions by removing the most clearly problematic items while preserving the intended distinction in demands. This procedure resulted in a 14-item lower-demanding form and a 12-item difficult form for use in the main analyses. Because the two refined forms differed in length, performance was indexed for each participant as the proportion of correct responses (i.e., number of correct responses divided by the number of retained items), thereby placing the two task versions on the same 0-to-1 metric and allowing direct comparison across forms.

To test whether the two FR tasks differed in difficulty, we also conducted one-way ANOVAs comparing the lower-demanding and higher-demanding task conditions on both objective performance and perceived task difficulty (i.e., “How difficult did you find the task?”, Likert response from 1 = “Extremely easy” to 10 = “Extremely hard”). Results showed that participants in the higher-demanding condition scored significantly lower on the FR task (M = 0.545, SD = 0.175) than those in the lower-demanding condition (M = 0.775, SD = 0.168), F(1, 240) = 107.918, *p* < .001, η^2^ = 0.310. In addition, participants in the higher-demanding condition perceived the task as significantly more difficult (M = 6.000, SD = 2.038) than those in the lower-demanding condition (M = 3.510, SD = 2.344), F(1, 240) = 75.408, *p* < .001, η^2^ = 0.239. Consistent with Zhou and Jia (2023), these findings support the distinction between the two task versions in both objective and subjective difficulty.

## 3. Results

Analyses were conducted using SPSS software (version 31). Descriptive statistics and correlations among variables are reported in Table 1.

For testing the study’s hypotheses we used a multiple regression approach (Aiken et al., 1991). A three-way moderated mediation analysis was modeled using PROCESS macro (Model 11; Hayes, 2018) with 95% confidence intervals and 5000 bootstrap samples. Continuous independent variables were mean-centered prior to the analysis. Before interpreting the regression models, the main assumptions of linear regression were examined. Normality of residuals was inspected through normal probability plots and residual distribution plots; linearity and homoscedasticity were evaluated by inspecting plots of standardized residuals against standardized predicted values; multicollinearity was assessed using tolerance and variance inflation factor (VIF) values; and potentially influential observations were examined using Cook’s distance. These diagnostics did not indicate relevant violations of model assumptions. Full diagnostic results are reported in the Supplementary Materials. Results of three-way moderated mediation analysis are presented in Table 2. The model predicting flow explained 30% of the variance (*R*^2^ = 0.303, *p* < .001), whereas the model predicting FR score explained 35% of the variance (*R*^2^ = 0.350, *p* < .001).

Consistent with a multiple linear regression approach (Aiken et al., 1991), promotion focus, prevention focus, FR task difficulty group (dummy coded: 0 = lower-demanding FR task; 1 = higher-demanding FR task), and their interactions (promotion × prevention, promotion × FR task difficulty, prevention × FR task difficulty, and promotion × prevention × FR task difficulty) were entered as predictors of flow. Flow was specified as the mediator, and the proportion of correct responses on the FR task was entered as the dependent variable.

Results showed that neither promotion focus, *b* = 0.263, *SE* = 0.142, *p* = .066, 95% CI [−0.017, 0.543], nor prevention focus, *b* = 0.165, *SE* = 0.095, *p* = .083, 95% CI [−0.022, 0.352], was directly significantly associated with flow. The promotion × prevention interaction was also not significant, *b* = −0.094, *SE* = 0.128, *p* = .464, 95% CI [−0.346, 0.158], nor was the promotion × FR task difficulty interaction, *b* = 0.045, *SE* = 0.216, *p* = .837, 95% CI [−0.380, 0.470]. The prevention × FR task difficulty interaction was also not significant, *b* = −0.259, *SE* = 0.153, *p* = .092, 95% CI [−0.561, 0.043]. By contrast, FR task difficulty group was significantly associated with flow, *b* = −1.170, *SE* = 0.125, *p* < .001, 95% CI [−1.417, −0.923], indicating that participants in the high-difficulty FR condition reported lower flow than those in the lower-demanding condition. Most importantly, the three-way interaction between promotion, prevention, and FR task difficulty was significant, *b* = 0.798, *SE* = 0.249, *p* = .002, 95% CI [0.307, 1.289].

**Figure 2:**
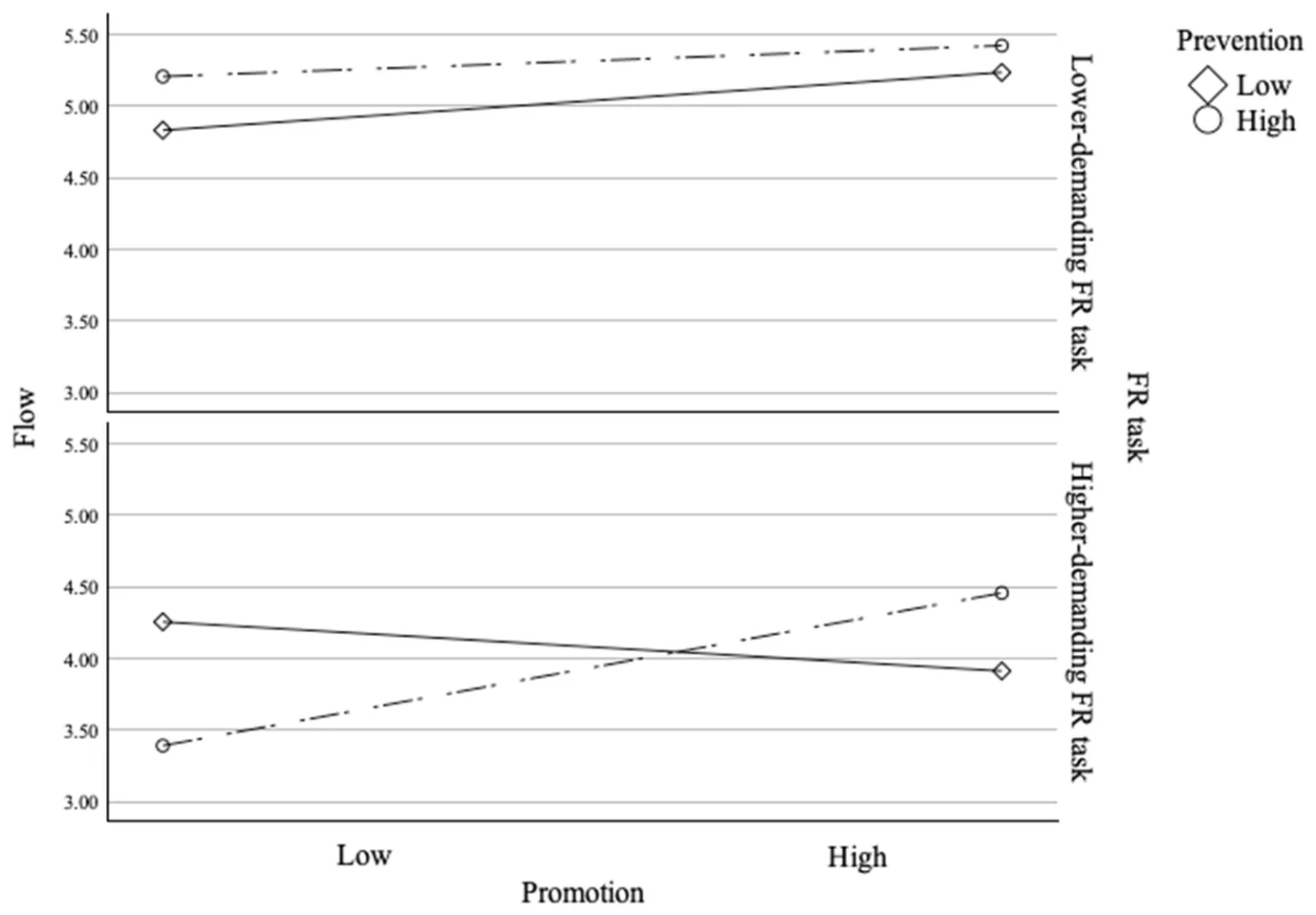
Simple slope analysis.

Simple slope analyses (Figure 2) showed that, in the lower-demanding FR condition, promotion was positively associated with flow when prevention was low, *b* = 0.343, *SE* = 0.171, *p* = .046, 95% CI [0.006, 0.679], but not when prevention was high, *b* = 0.183, *SE* = 0.187, *p* = .329, 95% CI [−0.185, 0.551]. In the higher-demanding FR condition, promotion was positively associated with flow when prevention was high, *b* = 0.906, *SE* = 0.243, *p* < .001, 95% CI [0.428, 1.384], whereas it was not significantly associated with flow when prevention was low, *b* = −0.291, *SE* = 0.245, *p* = .236, 95% CI [−0.774, 0.192].

Flow, in turn, was positively associated with a higher proportion of correct responses on the FR task, *b* = 0.110, *SE* = 0.010, *p* < .001, 95% CI [0.090, 0.129], whereas the direct effect of promotion on FR performance was not significant, *b* = −0.005, *SE* = 0.018, *p* = .809, 95% CI [−0.041, 0.032] when flow was in the model.

With regard to the moderated mediation test, the results did not fully support Hypothesis 1, whereas Hypothesis 2 was supported. Specifically, the conditional indirect effect of promotion focus on FR performance through flow was significant when prevention was high in the higher-demanding FR task condition, *b* = 0.099, BootSE = 0.027, 95% boot CI [0.049, 0.154]. In the lower-demanding condition, the conditional indirect effect of promotion on FR performance through flow at low prevention was in the expected direction but was not statistically significant, *b* = 0.038, BootSE = 0.021, 95% boot CI [−0.003, 0.080]. As expected, the indirect effect was also not significant when prevention was high in the lower-demanding condition, *b* = 0.020, BootSE = 0.015, 95% boot CI [−0.008, 0.053], nor when prevention was low in the higher-demanding condition, *b* = −0.032, BootSE = 0.028, 95% boot CI [−0.077, 0.034].

The index of moderated mediation was significant, index = 0.087, BootSE = 0.027, 95% boot CI [0.030, 0.135], indicating that the conditional indirect effect of promotion on FR performance through flow significantly differed across levels of prevention and task difficulty. Consistent with the indices of conditional moderated mediation (Hayes, 2018), the conditional indirect effect was not supported in the lower-demanding condition, index = −0.010, BootSE = 0.014, 95% boot CI [−0.037, 0.019], but was supported in the higher-demanding condition, index = 0.077, BootSE = 0.023, 95% boot CI [0.028, 0.118]. Overall, these findings partially supported Hypothesis 1, in that high promotion combined with low prevention was associated with greater flow in the lower-demanding condition, although the corresponding conditional indirect effect through flow was not statistically significant. Hypothesis 2 was supported, showing that high promotion combined with high prevention was indirectly associated with better FR performance through flow when the task was harder.

## 4. Discussion

Fluid intelligence has been conceived as the capacity to detect abstract underlying relationships, patterns, and rules relatively independently of previously acquired knowledge (Cattell, 1987). Fluid intelligence plays an important role in people’s lives, as it is associated with performance in several relevant domains, including mathematical skills (Green et al., 2017) and complex problem solving (Kyllonen et al., 2017). In the present study, fluid intelligence was assessed through a specific FR task (i.e., Raven-like matrices). This task requires relational reasoning under varying levels of complexity, with performance typically becoming slower and less accurate as relational demands increase (E. Ferrer et al., 2009). Despite the importance of studying possible correlates of fluid intelligence, there is still limited evidence concerning how motivational dynamics may shape performance in FR tasks. The present study aimed to address this gap by examining whether dispositional and state-like motivational factors could be associated with FR performance. Drawing on the integration of Regulatory Focus Theory (Higgins, 1997; Lee & Higgins, 2009; Scholer & Higgins, 2012) and Flow Theory (Csikszentmihalyi, 1990; Martin & Jackson, 2008), the results of the study confirmed that promotion and prevention orientations interact differently depending on the difficulty of the FR task, promoting higher levels of optimal engagement (i.e., flow). In turn, flow was found to be positively associated with higher performance in fluid intelligence test.

More specifically, and partially consistent with Hypothesis 1, the results showed that the interaction between high promotion focus and low prevention focus was associated with increased psychological flow in the lower-demanding FR task. This finding is consistent with a regulatory fit interpretation (Higgins, 2005; Lee & Higgins, 2009), according to which engagement is strengthened when the manner of goal pursuit is compatible with the characteristics of the task (i.e., how the task is processed). In particular, and in line with previous work (Keller & Bless, 2008), this fit may emerge when a relatively challenging task is approached with a focus on promotion. In such cases, a less demanding FR task enables people with a focus on promotion to achieve their desired goals of advancement, accomplishment and gain, pursued through eager strategies.

Conversely, when task demands are relatively low, high prevention may be less compatible with smooth and intrinsically rewarding engagement, given its stronger emphasis on security, non-losses, vigilance, and careful monitoring. Coherently, promotion was not significantly associated with flow when prevention was high, suggesting that the motives of the prevention orientation may be less functional in a task that does not require high vigilance to be solved optimally. However, Hypothesis 1 was not fully supported. Although promotion was positively associated with flow at low levels of prevention in the lower-demanding condition, the corresponding conditional indirect effect on FR performance through flow was not supported by the analyses. One possible explanation is that, although promotion facilitated flow under low prevention, the magnitude of this effect was relatively modest and therefore insufficient to produce a reliable downstream effect on FR performance. This pattern suggests that, although promotion may facilitate the experience of flow under favorable motivational conditions, such increases in flow may not have been sufficiently large to translate into measurable gains in performance when task demands were relatively low. In other words, the lower cognitive demands of the task may have reduced the extent to which variation in flow translated into measurable differences in FR performance. A second—related—explanation concerns the nature of the task itself. Since flow is more likely to emerge when skills and task demands are aligned (Csikszentmihalyi, 1990; Norsworthy et al., 2021), the lower-demanding task, which included a balanced assortment of items of varying difficulty, with most items falling under “medium” difficulty, may have provided favorable conditions for engagement for a broad range of participants. Consistent with this interpretation, the predicted values shown in Figure 2 indicate that flow was comparatively high across motivational profiles in the lower-demanding condition. Under these circumstances, the task itself may have provided favorable conditions for experiencing flow, thereby reducing the extent to which motivational orientations could account for additional variance in flow.

Hypothesis 2 was supported. Promotion focus was indirectly associated with FR performance through psychological flow when prevention was high rather than low in the higher-demanding condition. When the FR task is more demanding, the motivational contribution of both regulatory foci may support the use of strategies that are more compatible with the way the task needs to be processed. More complex FR matrices may require motives characterized by the complementarity of the two foci (i.e., Chen et al., 2017; Parker et al., 2014), enabling individuals both to advance toward a solution and to monitor potential errors. Under these conditions, and consistently with Keller and Bless (2008), flow may reflect the compatibility between the particularly hard-challenging nature of the task and the activated motivational orientations, in which advancement-related goals and security-related goals are jointly functional, together with their corresponding strategic modes of eagerness and vigilance. The results of the present study highlight the importance of the compatibility between person- and task-related characteristics in promoting task engagement (Barthelmäs & Keller, 2021) and supporting performance on fluid reasoning tasks. More broadly, the present findings extend recent applications of Regulatory Focus Theory to the domain of fluid reasoning, suggesting that motivational fit may represent one condition under which cognitive resources are optimally deployed (Nakkawita & Higgins, 2024). In the particular domain of fluid intelligence, performance in fluid reasoning tasks may depend not only on basic cognitive abilities but also on motivational factors that shape how individuals engage with task demands.

The present study also contributes to the literature on the association between flow and cognitive performance (Landhäußer & Keller, 2012) offering novel insights. Existing evidence suggests that flow is positively associated with cognitive performance, but the available findings remain largely correlational and do not yet allow firm conclusions about causal direction (Barthelmäs & Keller, 2021; Harris et al., 2023). Indeed, prior research on the association between flow and cognitive ability has been mixed. For example, Payne et al. (2011) found that cognitively demanding activities elicited higher levels of flow among individuals with higher fluid ability, but lower levels of flow among those with lower fluid ability, suggesting that the relation depends on the match between ability and task demands. Further, Ullén et al. (2012) found only weak or null associations between dispositional flow proneness and intelligence. Consistent with this background, the present findings suggest that the relation between flow and fluid intelligence may be understood not as a general direct link, but as a task-sensitive and motivationally conditioned process (Barthelmäs & Keller, 2021). In this sense, the motivational conditions under which fluid intelligence is assessed appear to represent an important factor to consider when cognitive abilities related to fluid intelligence are examined.

Limitations of the present study should be acknowledged, also raising opportunities for future research. The use of a convenience sample limits the generalizability of the findings. Future studies can replicate the present results in more diverse samples, including participants from different sociocultural contexts balancing sample distributions in terms of socio-demographic variables. Further, the direction of the relationships examined in the present study should be interpreted with caution. Although the literature suggests that flow may support task performance, including performance in cognitively demanding activities (Barthelmäs & Keller, 2021), the present design does not allow causal conclusions. In particular, it cannot be ruled out that higher FR ability may have facilitated the experience of flow, especially when the task more closely matched participants’ cognitive skills (Payne et al., 2011). Future longitudinal designs can test alternative or reciprocal pathways and studies based on the experimental induction of regulatory focus could help clarify the direction of the association between motivational orientations and flow experienced during task performance. In particular, prior research suggests that the accessibility of promotion and prevention focus can be situationally induced through procedures such as writing about aspirations versus duties, reflecting on past experiences, or exposure to gain/nongain versus loss/nonloss framing (Scholer & Higgins, 2012). The findings of the present study could also be strengthened by future studies that examine other variables that influence the relationship between self-regulatory foci and flow. For example, it would be interesting to examine the role of specific strategies and tactics, such as attentional patterns and problem-solving tactics, that people can adopt based on their respective foci. This would allow us to directly assess how the regulatory fit between ends and means influences flow and fluid intelligence. Finally, although the two task versions differed in objective and subjective difficulty, the present design does not allow for a complete evaluation of whether each condition represented an optimal balance of challenge and skill for participants. This distinction may be important for understanding the emergence of flow.

## 5. Conclusions

The present study concerns the distinction between dispositional and situational motivation and their joint relevance for fluid intelligence performance. Regulatory focus may be conceptualized as a relatively stable, trait-like motivational orientation, reflecting chronic sensitivities to distinct desired end-states that can coexist in different combinations within the same individual (Scholer & Higgins, 2012). On the other hand, flow is better understood as a state-like motivational experience that emerges contingently during task engagement and reflects the quality of individuals’ momentary involvement in the activity (Barthelmäs & Keller, 2021). Because research on motivational determinants of fluid intelligence remains limited, the present findings offer novel evidence that both dispositional motivational orientations and in situ motivational states may be relevant for understanding performance in cognitively demanding tasks. In this sense, the study extends current knowledge by suggesting that fluid intelligence may be shaped not only by cognitive resources per se, but also by the motivational conditions under which these resources are deployed.

## Figures and Tables

**Table 1:** Descriptive statistics and correlations.

Variable	Mean (SD)	1	2	3	Skewness	Kurtosis
1. Promotion	3.341 (0.588)				−0.381	0.18
2. Prevention	3.394 (0.850)	0.239 **			−0.213	−0.420
3. Flow	4.677 (1.115)	0.146 *	0.08		−0.458	−0.580
4. FR score	0.674 (0.206)	0.074	0.130 *	0.592 **	−0.716	−0.062

Note. FR score represents the proportion of correct responses (correct responses divided by the total number of items in the administered test version), so that scores from the two FR test versions were expressed on the same 0–1 metric. * *p* < .05; ** *p* < .001.

**Table 2:** Three-way moderated mediation analysis.

Predictors	Outcomes							
	Flow				FR Score			
	B	SE	*p*	95% CI	B	SE	*p*	95% CI
Promotion	0.263	0.142	0.066	−0.017; 0.542	−0.005	0.018	0.809	−0.041; 0.032
Prevention	0.165	0.095	0.083	−0.022; 0.352	–	–	–	–
FR task	−1.170	0.125	*p* < 0.001	−1.417; −0.923	–	–	–	–
Flow	–	–	–	–	0.110	0.010	*p* < 0.001	0.090; 0.129
Promotion × prevention	−0.094	0.128	0.464	−0.346; 0.158	–	–	–	–
Promotion × FR task	0.045	0.216	0.837	−0.380; 0.470	–	–	–	–
Prevention × FR task	−0.259	0.153	0.092	−0.561; 0.043	–	–	–	–
Promotion × prevention × FR task	0.798	0.249	0.002	0.307; 1.289	–	–	–	–

Note. FR task coding: 0 = lower-demanding FR task; 1 = higher-demanding FR task.

## Data Availability

Data can be requested and are available upon reasonable request.

## Supplementary Materials

The following supporting information can be downloaded at: https://www.mdpi.com/article/10.3390/jintelligence14080167/s1, Figure S1: PP-plots; Figure S2: Scatterplots; Figure S3: Wright-Map lower-demanding FR task; Figure S4: Wright-Map higher-demanding FR task; Table S1: Multicollinearity diagnostic; Table S2: Rasch analysis lower-demanding FR task; Table S3: Rasch analysis higher-demanding task.
