# Supplementary material for: Motivational Underpinnings of Fluid Reasoning: The Fit Between Motivational Orientation and Task Demands in Promoting Psychological Flow
Source: J Intell. 2026 Aug 1;14(8):167. doi: 10.3390/jintelligence14080167 (PMC13514357; doi:10.3390/jintelligence14080167)

Supplementary material

Regression diagnostic analyses

Regression assumptions were examined for both regression equations included in the model, namely the model predicting flow and the model predicting FR score. Overall, inspection of the normal P-P plots suggested that standardized residuals were approximately normally distributed. Scatterplots of standardized residuals against standardized predicted values did not reveal clear curvilinear or funnel-shaped patterns, supporting the assumptions of linearity and homoscedasticity. Cook's distance values ranged from .000 to .049 in the model predicting flow and from .000 to .073 in the model predicting FR score. In both models, all values were well below the conventional threshold of 1, indicating that no individual observation exerted undue influence on the model estimates. Finally, multicollinearity diagnostics (Table S1) indicated no evidence of problematic collinearity among predictors, with tolerance values ranging from .530 to .955 and VIF values ranging from 1.047 to 1.888.

Figure S1. PP-plots

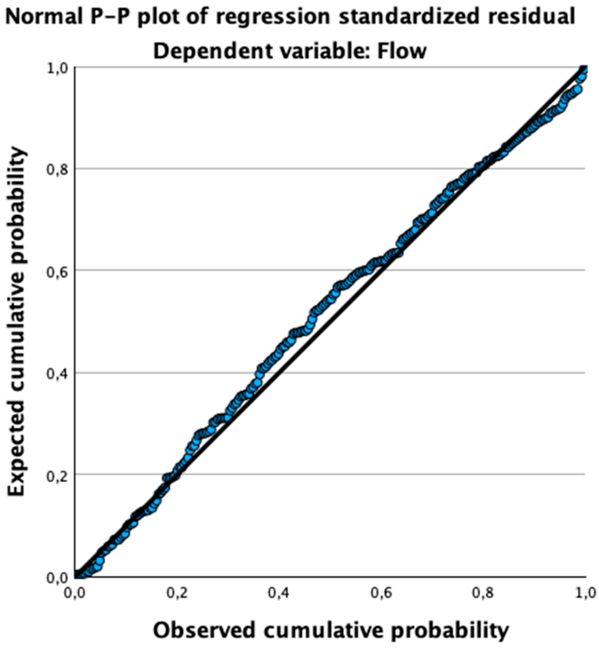

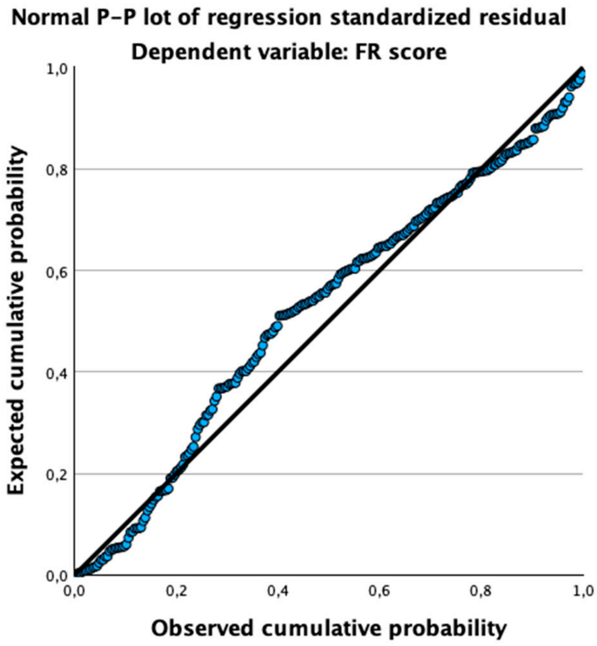

Figure S2. Scatterplots

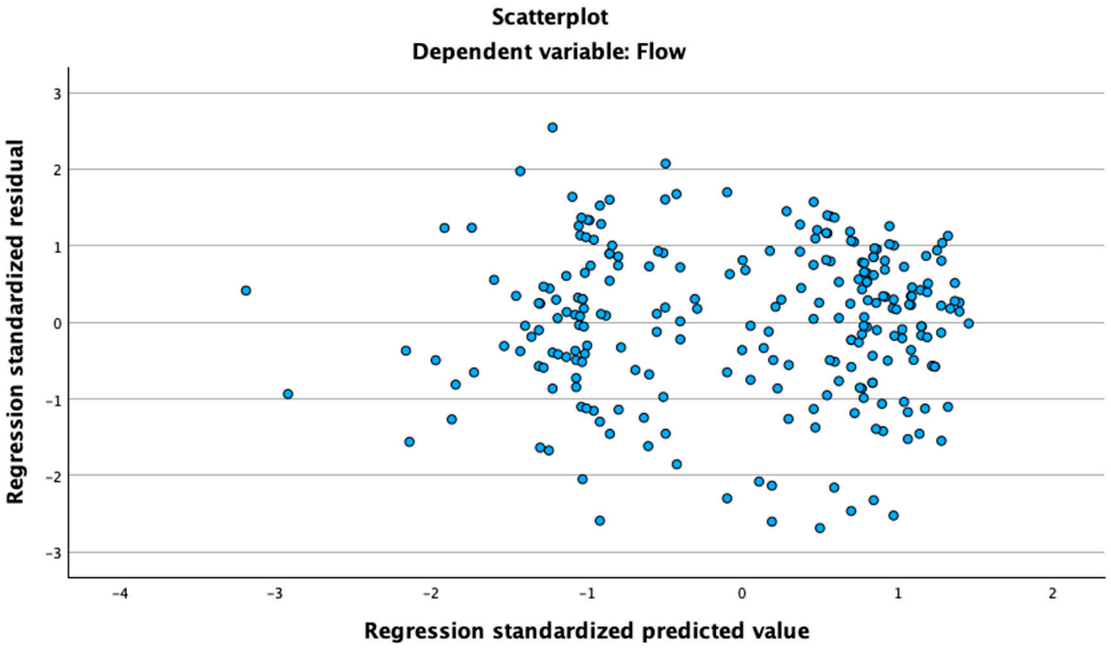

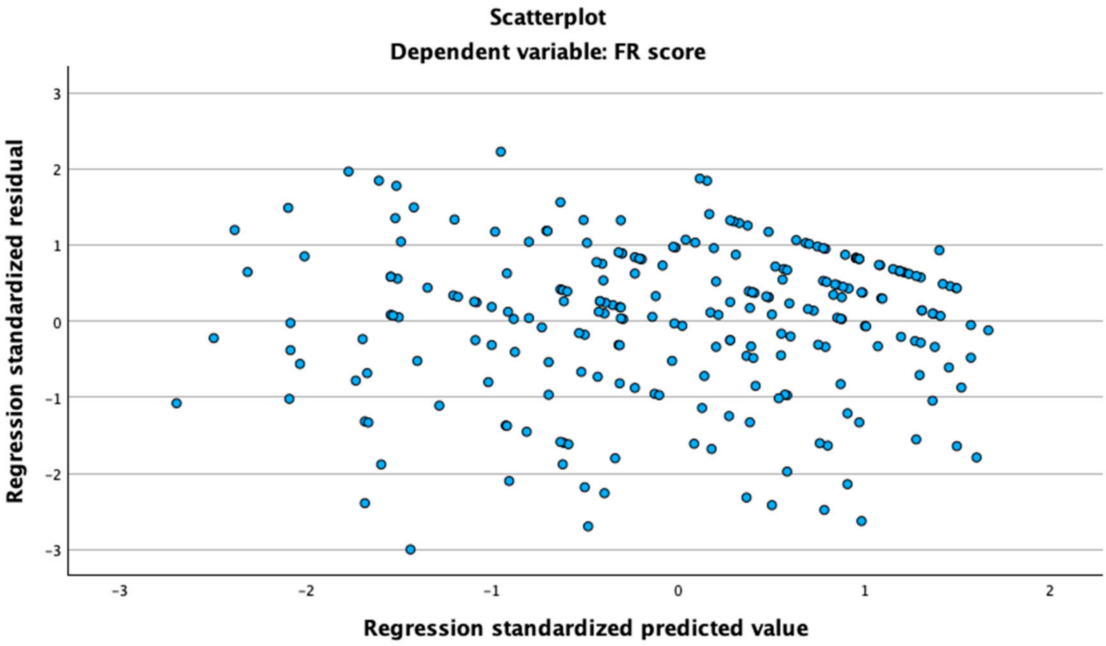

Table S1. Multicollinearity diagnostic

Predictors	Outcome			
	Flow		FR score	
	Tolerance	VIF	Tolerance	VIF
Promotion	0.530	1.888	0.979	1.022
Prevention	0.571	1.751	–	–
FR task	0.955	1.047	–	–
Flow	–	–	0.979	1.022
Promotion x prevention	0.718	1.393	–	–
Promotion x FR task	0.544	1.838	–	–
Prevention x FR task	0.575	1.740	–	–
Promotion x prevention x FR task	0.695	1.440	–	–

Rasch analysis

Table S2. Rasch analysis lower-demanding FR task

Item	Proportion	Infit	Outfit
item 1	0.985	0.884	0.199
item 2	0.868	1.072	1.064
item 3	0.118	1.144	1.546
item 4	0.860	1.023	1.017
item 5	0.699	1.112	1.175
item 6	0.838	1.139	1.355

item 7	0.875	0.982	0.823
item 8	0.721	1.068	1.188
item 9	0.765	0.902	0.810
item 10	0.669	0.980	0.990
item 11	0.824	0.905	0.916
item 12	0.949	0.853	0.589
item 14	0.868	0.811	0.633
item 15	0.816	0.921	0.904

Figure S3. Wright-Map lower-demanding FR task

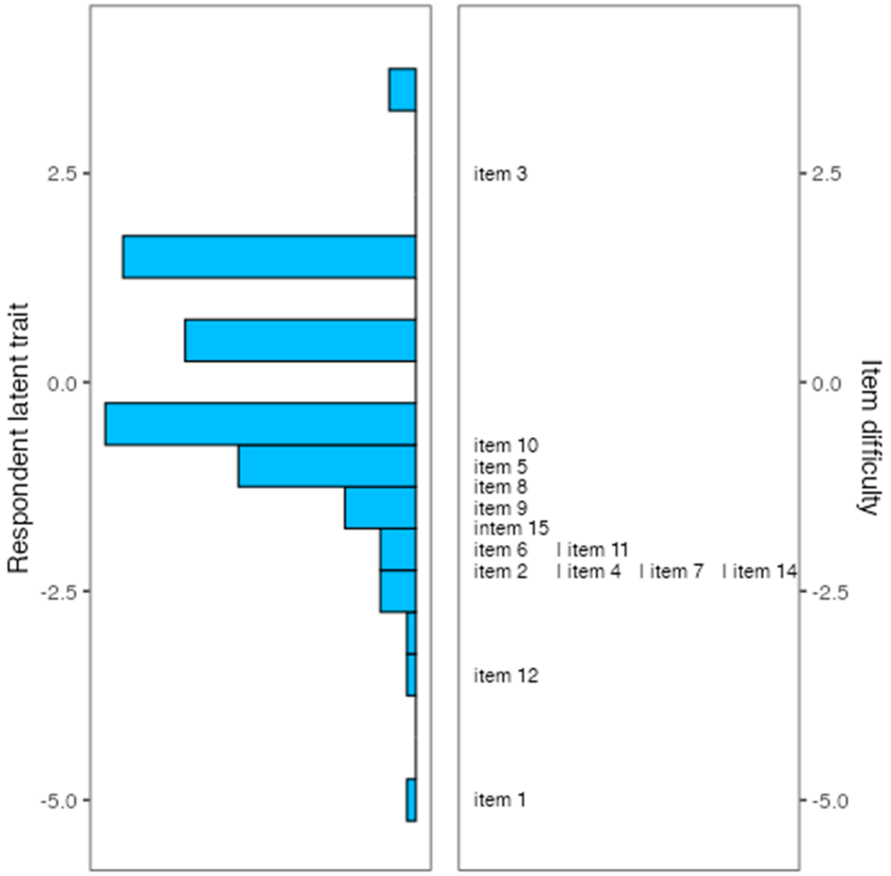

Table S3. Rasch analysis higher-demanding task

**Item**	**Proportion**	**Infit**	**Outfit**
Item 1	0.953	0.972	1.275
Item 2	0.953	0.910	0.882
Item 3	0.877	0.931	1.133
Item 5	0.585	0.973	0.962
Item 6	0.774	0.923	0.872

Item 9	0.651	1.018	1.059
Item 10	0.283	1.009	0.971
Item 11	0.434	0.948	0.933
Item 12	0.104	1.053	1.099
Item 13	0.443	0.971	0.944
Item 14	0.179	1.166	1.292
Item 15	0.302	1.019	1.056

Figure S4. Wright-Map higher-demanding FR task

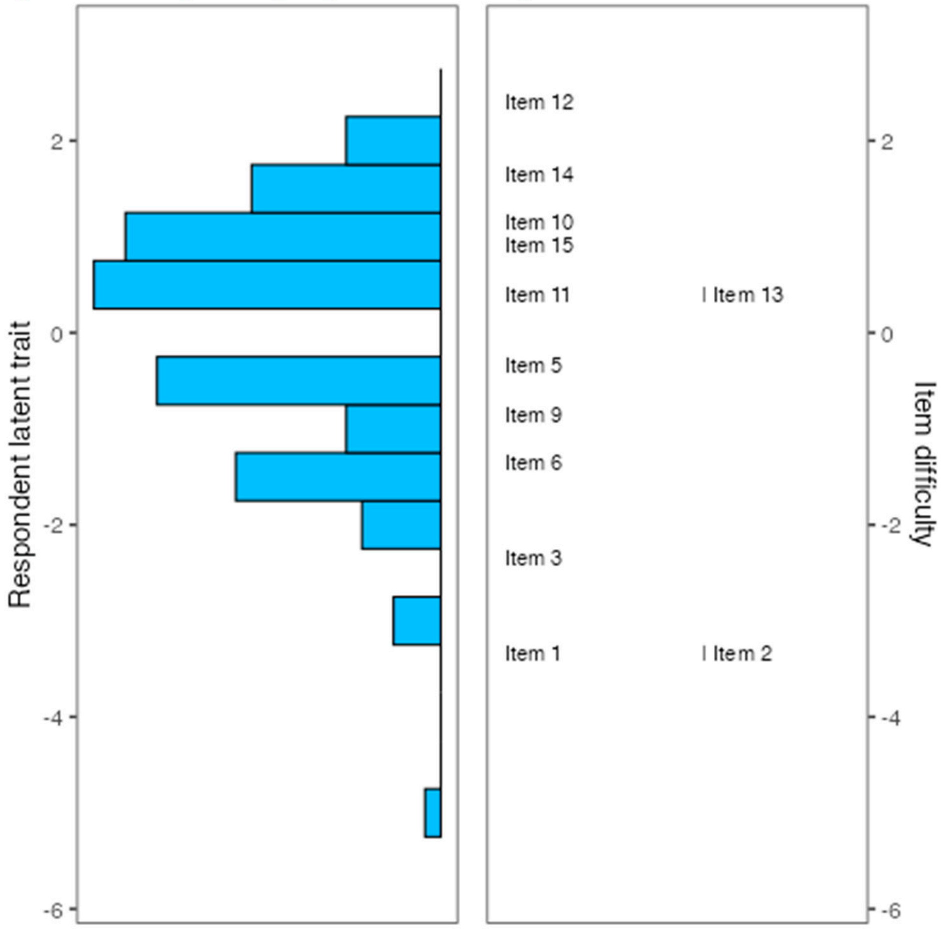

Supplement: Supplementary file 1 [file jintelligence-14-00167-s001.zip › jintelligence-4355607-supplementary.pdf]
